# A national data sharing solution for the prevention and treatment of obesity—a qualitative study of stakeholders’ needs

**DOI:** 10.1177/20552076241297740

**Published:** 2024-11-26

**Authors:** Anne Christenson, Joanna Uddén Hemmingsson, Ylva Trolle Lagerros

**Affiliations:** 1 Department of Medicine Solna, Clinical Epidemiology Division, 27106 Karolinska Institutet, Stockholm, Sweden; 2 Center for Obesity, Academic Specialist Center, Region Stockholm, Sweden; 3 Obesity Department, 59560 Capio StGoran's Hospital, Stockholm, Sweden; 4 Department of medicine Huddinge, H7, 27106 Karolinska Institutet, Stockholm, Sweden

**Keywords:** Prevention, disease, health informatics, general, obesity, lifestyle, connected devices, personalized medicine, self-monitoring, qualitative, studies

## Abstract

**Study objectives:**

Obesity-related health data is needed for studies and precision medicine, but existing registers, medical chart systems, and digital platforms are seldom compatible. Before creating improved ways of sharing health data, this study aimed to gather opinions, experiences, and wishes from stakeholders that may use obesity-related health data: healthcare, researchers, people with overweight or obesity, the pharmaceutical industry, and IT-specialists.

**Methods:**

We performed semistructured interviews with 28 stakeholders and analyzed qualitative text data with inductive content analysis. We grouped the suggested parameters in categories.

**Results:**

Time efficient data entering was perceived crucial. Access to health data was important to all participants. Some parameters, such as age, BMI, and sex were requested by all stakeholders. Other data were stakeholder specific, such as population-specific laboratory references, suggested by healthcare professionals only. For people with overweight or obesity, ability to share data with healthcare staff about fitness level or previous weight loss attempts, was important.

**Conclusion:**

The results from this study can be used in the design and implementation of a national health data sharing solution that may be used for precision healthcare use and to evaluate and guide obesity treatment and preventive measures. Data parameters requested by all populations, such as BMI, sex, and age, should be prioritized when designing a data solution. Ability for individuals with overweight or obesity to share health data, may improve healthcare appointments and reduce weight stigma.

- **What is already known on this topic**—Improved ways of accessing and sharing obesity-related health data is needed for evaluation, research studies, and precision medicine. However, little is known about stakeholders’ experiences and wishes.
- **What this study adds**—Herein, five groups of stakeholders suggest their primary parameters to be included in a future data sharing solution.
- **How this study might affect research, practice, or policy**—Suggested parameters from this study should guide the design of future data solutions.

## Introduction

The prevalence of obesity is increasing in Sweden as well as globally.^1^ Meanwhile, preventive interventions are insufficient and obesity care is undersized and unequal.^2^ To counteract the rampant increase in obesity prevalence and its sequelae, several international guidelines for obesity treatment have been released during the last decade.^3–5^ The Swedish Ministry of Health published national guidelines for obesity treatment in 2022.^6^ However, tracking implementation and assessing the impact of guidelines is challenging due to the lack of large scale, up to date, real-world health data.

The world is becoming increasingly digitalized. This includes healthcare, where patients, healthcare staff, medical researchers and the pharmaceutical industry all want access to digital medical data, each group for their specific needs. Likewise, public health actors rely upon population health data when designing preventive measures and guidelines. However, policies about medical treatment or prevention are often based on data that is collected retrospectively, or gathered from small study populations with specific inclusion criteria. As a result, decision-making may be based on data that is already outdated or incomplete.

Though vast amounts of health data are constantly being entered into various systems, data may be difficult to access across stakeholders, as existing registers, medical chart systems, and digital platforms are seldom compatible. For decades, policy analysts have suggested that multiinstitutional sharing of data from electronical health records presents an important opportunity for advancing population health.^7^ As early as 2008, an American study found that aggregating health data from larger population samples, by sharing electronic health record data from multiple institutions, was useful for the surveillance and study of childhood obesity.^7^ Still, in 2023, the World Health Organization (WHO) described that among the many challenges in tackling obesity are the fragmentation of care and insufficient collaboration between sectors and settings.^8^ The WHO report goes on to suggest increased “collaboration between organizations working toward a common goal by monitoring progress through indicators, and other monitoring tools and practices.”^8^

To facilitate an effective use of existing data, we need new ways to collect, process, and feedback health data to improve disease preventive measures and treatments, as well as to evaluate their implementation. A new digital data solution where structured medical data can be shared in real time, in a way that is safe and ensures integrity, would be preferable.

An initiative to develop such a digital data solution has been taken by the Swedish Association for the Study of Obesity (SASO). SASO is a part of the European Association for the Study of Obesity (EASO)—a federation of professional membership associations from 36 European countries. SASO represents the Swedish obesity community, promoting action through collaboration in advocacy, communication, education, and research. SASO collaborates with the Ministry of Health and other health authorities in Sweden, such as the Swedish Association of Local Authorities and Regions, as well as the National System for Knowledge-driven Management within Swedish health care, but also with the patient’s organization Obesity Sweden. All of these organizations support the advancement of a digital data solution for obesity care.

To develop such a digital data solution in a user-friendly way, it is important to first assess the experiences, opinions and wishes from relevant stakeholders. The purpose of this study was therefore to identify and describe (1) what relevant stakeholders need in terms of structured obesity-related health data, (2) how stakeholders would like such data to be entered and accessed, and (3) to assess their reasoning behind their wishes.

## Methods

### Study design

Our goal was to assess stakeholders’ subjective needs, views, experiences, and wishes. Furthermore, we aimed to present the requested parameters as well as the stakeholders’ reasoning behind their choices. Therefore, we chose a qualitative descriptive approach^9,10^ and collected data through semistructured, in-depth interviews.^9,11^

### Setting

In Sweden, healthcare is publicly funded, and specialist treatment is available to anyone who meets the referral criteria, regardless of their financial situation. The largest regions have specialist obesity treatment clinics, while most regions lack such facilities. During the data collection period, available antiobesity medications were naltrexone/bupropion, orlistat, and glucagon-like peptide-1 agonists.

### Study population and selection criteria

We identified the following five stakeholder populations who may either construct, use, or who have experience from sharing health data:

1. Healthcare professionals from different regions with experience of treating patients with obesity.
2. Representatives from academia conducting obesity research.
3. Individuals from different regions with overweight (BMI 25 to 29.9 kg/m^2^) or obesity (BMI ≥30 kg/m^2^).
4. Representatives from the pharmaceutical obesity treatment industry.
5. IT specialists with a focus on health data.

Among healthcare professionals we recruited participants from different clinical occupations commonly found in obesity treatment teams (physician, nurse, dietician, and physiotherapist). To attain variation in participants’ background, we used a strategic selection of participants based on characteristics.^11^ A variety of ages in each target population was considered important, as there may be differences between younger and older participants in attitudes toward sharing digital information. The inclusion of participants of different ages, also yielded a range of clinical experience in the subgroup of healthcare professionals. Among individuals with BMI ≥25 kg/m^2^, we aimed for a variety of BMI classes, as well as experience from having undergone bariatric surgery. Recruitment continued until the research group decided that sufficient interview data was collected from each group of stakeholders to answer the research questions (Table 1).

**Table 1.** Research questions.

1. What do stakeholders consider important regarding what data to include in a data solution? 2. What do stakeholders consider important when developing a new data sharing solution? 3. What are stakeholders’ experiences regarding advantages and disadvantages with existing data systems and tools? 4. How would stakeholders wish to enter, access and use health data? 5. How do stakeholders believe that increased access and usage of health data may affect the prevention and treatment of obesity?

### Data collection

Participants were interviewed via video or telephone during the years 2021–2023, using a semistructured interview guide (Supplemental Material S1). Interviews were recorded digitally. Data about age was collected from all participants. Information about height, weight and whether the person had undergone bariatric surgery was collected from the population with BMI ≥25 kg/m^2^.

### Recruitment

To reach a purposeful selection of participants with relevant knowledge, experience, and/or characteristics, this study combined strategic, as well as snowball sampling.^11^ This meant that eligible participants could suggest other people with relevant knowledge or characteristics to be interviewed. Participants with a BMI >30 kg/m² were recruited through specialist obesity treatment clinics and the national patient union's digital newsletter. The other stakeholder groups were reached via email, face-to-face interactions at scientific obesity meetings, or through suggestions from other participants. The principal researcher (AC) provided willing and eligible individuals with study information, obtained informed consent and scheduled them for an interview. Interview data was analyzed in parallel with data collection. The authors had regular meetings to decide whether the data retrieved so far was sufficiently rich to answer the research questions, or whether more participants should be interviewed.^9,11–13^ We determined that additional interviews would be redundant when no new information emerged in the last three interviews, reaching this point after conducting 26 interviews. However, two more participants were recruited to increase the number of younger voices in the material.

### Ethical considerations

The first author (AC) is a cognitive therapist with vast experience from obesity care and from qualitative studies. She conducted all interviews, ensuring that research participants felt safe and comfortable in openly sharing their thoughts about the research topic. All participants provided informed consent and agreed to have their interviews audio recorded.

### Analysis

Interview data was analyzed using manifest inductive qualitative content analysis.^14^ To reduce researcher bias and increase trustworthiness, two researchers were involved in the analysis.^15^ While reading the transcribed interview recordings repeatedly, relevant statements, such as those regarding what data to include in a data sharing solution, where highlighted and labeled with a code, formulated to describe the meaning of the statement, while staying close to the text.^9,14^ Both authors discussed coding discrepancies and agreed on their definitions during the iterative process of formulating codes.^9^

Subsequently, codes such as “presence of knee arthrosis” or “providing a diagnosis” were merged into subcategories such as: comorbidities or obesity diagnosing. In the next step subcategories were grouped together and four main categories were formulated to contain measurable data parameters: (1) Influenceable conditions, (2) Activities that are carried out, (3) Influenceable health parameters, and (4) Noninfluenceable patient characteristics. Text data about stakeholders’ experiences, wishes and usage of data were analyzed per subgroup to be able to describe stakeholder-specific details.

#### Trustworthiness

To attain rich, appropriate, and well-saturated data that produces trustworthy findings we used purposeful sampling, an experienced interviewer, and structured steps in our analysis.^16^ Researcher triangulation was used during the analysis to add to credibility.^17^ Apart from sampling participants with relevant experience, we also recruited participants from different regions and clinics, as well as with different background characteristics to increase transferability. To further increase trustworthiness, quotes were chosen to confirm how findings were grounded in the original data.^16^

#### Presentation of the findings

The goal of this study was to present findings that could guide the development of future data solutions. To facilitate this, data regarding desired parameters was organized into comprehensible summary tables to enable interpretation.^9^ The tables are followed by text descriptions where data is categorized, to provide a deeper understanding of the choices made by each stakeholder group.

## Results

### Study population

During recruitment and interviews, seven participants displayed characteristics that fit into more than one population of stakeholders, for example a researcher who was also a healthcare professional. They were allowed to share their opinions from different stakeholders’ views. Interviews lasted between 7 and 65 min (mean 38 and median 34 min), where the shortest interview was with a participant from the group of ≥25 kg/m^2^ who chose to only elaborate on a few questions. Table 2 shows the baseline characteristics for participants in each group of stakeholders.

**Table 2.** Participant characteristics.

Subgroup	*n*	Women, *n*	Mean age in years (range)	Mean BMI (range)
Healthcare professionals^a^	8	5	48 (31–57)	–
Academia^b^	5	5	45 (26–57)	–
Individuals with overweight or obesity^c,d^	16	10	51 (22–65)	36 (26–54)
Pharmaceutical obesity treatment industry	3	1	48 (41–56)	–
IT specialists with a focus on health data	4	0	55 (48–62)	

BMI: body mass index.

^a^ Included professions: physiotherapist *n* = 2, dietician *n* = 2, medical doctor *n* = 3, and nurse *n* = 1.

^b^ Researchers or research assistants.

^c^ Ten participants had a BMI of ≥30 kg/m^2^.

^d^ Four participants had undergone bariatric surgery.

### Parameter needs

Data about height, weight, sex, and age were desired by all groups. Parameters requested from more than one group of stakeholders, though for different purposes, are found in Table 3. For example, data on exercise frequency was desired by academia as a way of assessing physical activity in the population. Meanwhile, people with obesity also wanted healthcare providers to see how much they exercise, hoping that this would prevent unsolicited advice regarding physical activity.

**Table 3.** Parameters to be included in a data sharing solution, desired by at least two groups of stakeholders.

**Influenceable conditions**	
Which clinics offer obesity preventive/treatment interventions in primary care?	
What components are included in the preventive/treatment interventions?	
	-Dietary support
	-Physical activity support
	-Psychosocial support for lifestyle changes?
Which professions are available in the team?	
Is there a way for patients with obesity to rate how they perceive the approach and treatment at a clinic?	
**Activities that are carried out**	
Anthropometric measuring	
	-Are height, weight and waist measured?
Obesity diagnosing	
	-Is obesity diagnosed?
	-What medical examinations underpin the obesity diagnosis?
Offered interventions	
	-Are interventions offered?
	-How many patients utilize each intervention and to what extent?
Weight loss drug treatment	
	-What kind of drugs is prescribed?
	-Who suggests them, at which BMI?
Patient adherence	
	-What supportive actions are taken?
	-What is the follow-up frequency?
	-Is compliance measured?
Bariatric surgery	
	-Are referrals written for obesity surgery?
	-At which BMI?
	-Who suggests bariatric surgery (the patient or which health profession)?
**Influenceable health parameters**	
	-Health-related quality of life
	-Weight
Comorbidity	
	Diabetes type 2, polycystic ovary syndrome, cardiovascular disease, hypertensive disease, sleep apnea, dyslipidemia, infertility, musculoskeletal pain
Physical activity level	
	-Steps per day
	-Exercise sessions per week
	-Cardiovascular fitness level
**Noninfluenceable patient characteristics**	
	Height
	Sex
	Age

Meanwhile, other parameter suggestions were mentioned by only one group of stakeholders (Table 4).

**Table 4.** Parameters to be included in a data sharing solution, specifically suggested from each individual group of stakeholders.

**Healthcare professionals**	
Influenceable conditions	
	Risk tool to show different future scenarios to the patient
Activities that are carried out	
	Are individual dietary energy levels calculated and prescribed?
	Are liquid low energy diets used, and for which patients?
	Are food registrations used and analyzed?
	Is physical activity on prescription used?
	Are aspects of mental well-being measured?
Influenceable health parameters	
	Population-specific lab data for people with obesity
**Academia**	
Influenceable conditions	
	Data from food stores about purchased food items
**Individuals with overweight or obesity**	
Influenceable conditions	
	Is cognitive behavioral therapy available?
	Rating of individual doctors or clinics to find obesity friendly clinics
**Pharmaceutical obesity treatment industry**	
Influenceable conditions	
	Are healthcare staff educated about weight loss drugs and obesity treatment?
	Are there routines for which patients are offered weight loss drugs?
Activities that are carried out	
	To what extent are patients offered psychotherapy?
	How many patients are referred to sleep apnea examination?
IT specialists with a focus on health data*	
*This subgroup was not asked about specific parameter wishes. Instead, they contributed with their experiences regarding data solutions, which are presented in the running text.	

An in-depth analysis of experiences, and suggestions on why and how data should be entered and accessed, is presented in detail below, per study population.

### Healthcare professionals

#### Time efficiency

The main concern for healthcare providers was time efficiency. Preferably, there would be standard templates, and data should be downloaded automatically from the medical chart. During manual data entry, they requested a tick box, for example “smoker,” without having to add number of cigarettes per day. Such conditional answers were perceived as frustrating, time-consuming, and leading to inaccurate entry of data.

*Not many physicians ask their patient exactly how many cigarettes they smoke per day (during a standard consultation). So instead, you guess, or just randomly press ‘smoker’ or ‘nonsmoker’ (to be able to continue data entry). Hence, existing data about smoking and alcohol is completely useless. Still, vastly used in research.* Participant #7

Meanwhile, other health professions wanted the ability to specify, in more detail, what advice had been provided, for example regarding diet.

*…whether it includes a diet with a calory restriction, or a very low calory diet with meal replacement products.* Participant #1

#### Treatment tools

Health professionals thought that risk calculation programs would be helpful where they could enter patients’ anthropometric data and receive an estimate of future health development. To improve precision treatment, they wished to have access to laboratory reference values from patients with obesity, to avoid unnecessary investigations based on only seemingly pathological values.

*For example, a high erythrocyte sedimentation rate (ESR) is common among people with obesity, which may render a number of unnecessary investigations, /…/ So, if we had laboratory references for this particular population then we would not have to expose them to a lot of unnecessary tests.* Participant #7

It was suggested that people with overweight or obesity could enter health parameters in a health profile to share with healthcare, for example ahead of a healthcare visit. Another common wish from healthcare providers was to be able to access a digital portal with continuously updated clinical guidelines.

### Academia

#### Improved research validity

Researchers would like to have automatic transfer of health parameters from medical charts, as well as from self-reports in studies, into a register. They valued access to both regional and national data for the ability to make large scale comparisons.

*We need to not only access data, but also enable data coverage which is the major lack in registry data. Today we only have full data coverage from specific interventions for cardiovascular diseases and perhaps also regarding cancer mortality. But if you look at endemic diseases like chronic obstructive lung disease, obesity, or diabetes, which take 50% av all healthcare cost, the data coverage is so bad that the precision of advice to healthcare is affected.* Participant #11

People in research highlighted that access to obesity related health and weight data from primary care would improve the definition of what “usual care” really means in randomized controlled trials. In addition, they would like data from registries to be accessible for healthcare staff as well, and possibly also feedbacked to individuals with obesity. To enable this interoperability, researchers brought forward the necessity to have a national common nomenclature in medical charts to simplify mapping, overviews and to make texts machine readable.

### Individuals with overweight or obesity

#### Story sharing for improved treatment

The main wish from individuals with a BMI of 30 kg/m^2^ and above, was to receive a respectful and less stigmatizing treatment, both in society and within healthcare.

*Sometimes I feel as if I am looked upon as ill only because I am fat./…/ It is important to find a more positive and more encouraging way of approaching patients with a high BMI rather than to scare and judge them.* Participant #17

One suggested way of achieving a better treatment was to have the possibility to voluntarily share data with healthcare professionals about previous weight loss attempts. To be able to share exercise data from private health apps was suggested as a mean to lower the risk for healthcare staff to automatically assume that a patient with a large body is physically inactive.

*If I have entered a lot of data (about exercise) in apps or medical journals, I would like to be able to share those data with whomever I want.* Participant #16

*…not having to tell the same story (over and over again), it would be nice if they (healthcare providers) could access all the information that they need (from other data systems).* Participant #20

Ability to follow other parameters than weight, including comorbidities (or absence thereof) were believed helpful in accepting one's own weight. It was described that this could remove the stress and anxiety about weight loss and instead facilitate focusing on health in a more holistic way.

*…to include other health parameters such as oxygen uptake, or comorbidities /…/ because I am ‘morbidly obese’ according to BMI but I am not ill. You can have a large body without being ill.* Participant #16

Participants with obesity wanted access to other people's stories about body, self-image and how they felt treated by others. Such data could reveal what people with different BMI-levels may experience in society. They also wished for the possibility to anonymously compare their weight with other people and to see photos showing what real bodies with excess skin look like after large weight loss.

*… statistics about weight and BMI and I also believe it would be interesting to gather some kind of aspects regarding self-image and how you perceive the way society looks at you at a certain weight or to what extent you feel accepted by society.* Participant #21

#### Reliable facts source

Furthermore, participants with overweight or obesity wished for a reliable source with facts about weight-related health, valid dietary advice, and healthy recipes. They wanted a digital portal with information about weight loss drugs, as well as which centers provide medical or surgical weight management programs.

*Everything you may ever need is out there if you google it, but the problem is that it is hard to filter, there is too much and everyone can claim to be an expert based on none, or unreliable scientific sources.* Participant #17

Both participants with overweight and with obesity described digital applications as helpful in supporting lifestyle changes, for example applications where you register what you eat, or mobile phone pedometers. Some wished for apps with reminders, for example for taking vitamin supplements post bariatric surgery.

#### Comfortable with sharing deidentified data

None of the individuals with BMI ≥25 kg/m^2^ expressed worries about integrity or feared that sharing data may be negative. Instead, they urged that researchers must be allowed access to individuals’ deidentified health and treatment data so that society and healthcare professionals gain increased knowledge, and thus increased understanding, about the risks and treatment of overweight and obesity.

*I have been a voluntary experimental guinea pig in several trials or investigations and I think it is necessary to attain more knowledge /…/All data needs to be accessible in one place instead of being stored in too many different systems.* Participant #15

*I have no problem with it (sharing health data). As long as the data is deidentified it does not matter.* Participant #20

### Pharmaceutical obesity treatment industry

#### Multisource real-world data

Representatives for the pharmaceutical industry wished to gain access to both regional and national real-world data about obesity prevalence, obesity treatment efforts, and antiobesity medication use in primary care. They wished for a digital platform that collect and distribute data from multiple sources, and where healthcare, academia, industry, and individuals can both create and share data. It was mentioned that a new solution should be designed as an interface where already existing data solutions and registers must be interoperable. To generate prospective data, it was suggested that each patient fill in a short standard form before every visit to their general practitioner, which could include, for example self-reported weight and weight loss drug use.

*Better follow-up is needed to ensure that the right patient receives the right treatment*. Participant #10

### IT specialists with a focus on health data

#### Immediate raw data access

Participants from the IT-sector did not know of any system in use with the ability to both enter and access obesity related data to and from all stakeholders, including individuals with overweight or obesity. They highlighted the need for a common terminology, and a process where humans do not interpret the data before it is entered in to the system, that is only raw data is collected.

*A dream would be to keep a data point as a data point, because a lot of data (in healthcare) is gathered, but it is often entered in medical records as text. Now everybody is talking about how we must ‘open’ the medical records (for research) but the problem is that data is already biased. We need to catch data already at the primary source.* Participant #24

It was mentioned that a solution needs to gather data and make it accessible at the same time, unlike existing quality registries where data become available only after a certain time or processing.

*…unlike quality registries, which are made up of a series of ‘snapshots’, entered into the system by someone when they have the time.* Participant #25

Participants also suggested that lawyers are consulted in the creation of a digital solution to ensure that legal aspects are considered. Depending on where and how data is stored, different laws apply.

## Discussion

This study identified and described what five groups of stakeholders would like in terms of health and weight data to improve interventions for weight-related health. A recent Australian review identified a comprehensive list of social, biomedical, behavioral, environmental, and commercial determinants of health relevant to obesity prevention.^18^ Meanwhile, the review also highlighted the need to identify and describe specific indicators that can inform decisions. Our results suggest that using measurable indicators as primary parameters would be beneficial for stakeholders from healthcare, academia, individuals with overweight or obesity, the pharmaceutical obesity treatment industry, and IT-specialists with a focus on health data.

A solution that enables individuals to enter, access, and share their health data themselves was requested from all stakeholders. More and up-to-date national population data could be useful when comparing interventions and regions and thus teach us more about obesity prevalence and treatment results. In the future, “big data” could facilitate individualized risk assessments and form the basis for developing a so-called “digital twin” which can raise awareness in an individual of what steps they could take to improve health.^19^ This would allow for each individual to be given the opportunity to participate in the creation of their own healthcare. Improved access to data trends, for example weight change in a population, could also facilitate disease prevention and health promotion activities.

In research, the term “usual care” is used to describe the intervention that study participants in a control group receive. However, the contents of usual care may vary substantially between clinics or regions. Furthermore, existing medical chart templates and questionnaires often use conditional questions in their aim to assess more detailed answers and higher quality data. The results from this study indicate that conditional questions may in fact render less accurate results. With easier access to healthcare data, based on optimized questionnaires, future studies may deliver more accurate comparisons and results.

As of today, to our knowledge, there is no existing national register or data solution that covers the needs and purposes of all stakeholders. Earlier studies of health data sharing systems such as the Colorado BMI Monitoring System, demonstrated the feasibility of sharing electronical health record data across multiple regional healthcare organizations for public health surveillance and research purposes.^20^ However, they did not include the possibility for patients to enter and share data.

In the present study both researchers and IT specialists suggested that a new digital solution should facilitate access to raw and unbiased data. Furthermore, they pointed out the need of a common national terminology to create so called semantic interoperability^21^ and machine-readable medical lexicons. Moreover, for easier implementation, any additional manual data entering must be minimized, as earlier studies also reported that retrieving data manually from electronical records was time consuming.^7^ A common template for medical charts, as suggested by healthcare professionals, may need to be created for primary care, as well as for medical and surgical specialist clinics.

The participants with a BMI ≥25 kg/m^2^ in this study seemed unconcerned about sharing their data. In fact, they suggested that shared health data may help prevent stigmatizing views. This is an important notion, as people with obesity are vulnerable to societal stigma and discrimination.^22^ However, we need clear regulations and ethical standards to ensure that inappropriate data sharing is precluded.^23^ A conceptual model for how to deal with technical and legal issues by enabling patients to grant permission to share real-world data to different stakeholders has been proposed in an action research study for cystic fibrosis care.^24^ Their model may be applicable in obesity care as well.

### Methodological considerations

A strength of this study is the varied backgrounds in the interviewees. This increases the transferability of the results to other populations. The relatively high mean age among participants may be seen as a weakness as future data solutions should appeal also to younger people. However, since we chose to include knowledgeable informants, for example physicians that were specialists in obesity care, and IT-specialists with long professional experience, it was reasonable that our participants were middle aged and above.

As the interviewee group is made up of a small sample, there may be more suggestions than the ones discussed here. Nevertheless, the results can establish that the wishes and needs presented in this study exist within the populations.

## Conclusions

The stakeholders in this study described that an effective health data solution should collect data directly from individuals and from healthcare, analyze data in real-time and feedback data to healthcare professionals, academia, pharmaceutical obesity treatment industry, and to individuals with overweight or obesity, for precision healthcare use. In a virtuous circle, more data input from individuals can improve predictions and subsequently the individual may benefit from more precise healthcare and less weight stigma. The results from this study can be used to influence the design and implementation of a data solution where data parameters that were requested by all populations, such as BMI, sex, and age, are proposed to be prioritized.

## Supplemental Material

sj-docx-1-dhj-10.1177_20552076241297740 - Supplemental material for A national data sharing solution for the prevention and treatment of obesity—a qualitative study of stakeholders’ needs Supplemental material, sj-docx-1-dhj-10.1177_20552076241297740 for A national data sharing solution for the prevention and treatment of obesity—a qualitative study of stakeholders’ needs by Anne Christenson, Joanna Uddén Hemmingsson and Ylva Trolle Lagerros in DIGITAL HEALTH

sj-docx-2-dhj-10.1177_20552076241297740 - Supplemental material for A national data sharing solution for the prevention and treatment of obesity—a qualitative study of stakeholders’ needs Supplemental material, sj-docx-2-dhj-10.1177_20552076241297740 for A national data sharing solution for the prevention and treatment of obesity—a qualitative study of stakeholders’ needs by Anne Christenson, Joanna Uddén Hemmingsson and Ylva Trolle Lagerros in DIGITAL HEALTH
