# Supplementary material for: A national data sharing solution for the prevention and treatment of obesity—a qualitative study of stakeholders’ needs
Source: Digit Health. 2024 Nov 26;10:20552076241297740. doi: 10.1177/20552076241297740 (PMC11590157; doi:10.1177/20552076241297740)
Supplement: sj-docx-1-dhj-10.1177_20552076241297740 - Supplemental material for A national data sharing solution for the prevention and treatment of obesity—a qualitative study of stakeholders’ needs [file sj-docx-1-dhj-10.1177_20552076241297740.docx]

Supplement 1:

**Interview guide**

(additional questions may be asked to probe topics that the participant shares)

1. What are your experiences regarding the use of digital tools or health data sharing solutions?
   1. Advantages and disadvantages?
   2. What could be done better?
2. What data would you consider important to access with the purpose to improve care and treatment of people with overweight or obesity?
3. What data would you consider important to access with the purpose to prevent obesity?
4. How, by whom, and when would you prefer health data to be entered?
5. What are your opinions on how aspects of integrity, usability and interactivity should be accounted for when sharing health data?
6. Is there anything else you would like to add that I have not asked you about?
